# Effects of group size on movement patterns and clustering dynamics in rats

**DOI:** 10.1093/oons/kvae005

**Published:** 2024-04-02

**Authors:** Marie-H Monfils, Michael Pasala, Cassidy Malone, Laura Agee, Rheall Roquet, Lawrence Cormack

**Affiliations:** Department of Psychology, The University of Texas at Austin, 108 E. Dean Keeton Stop A8000, Austin, TX 78712-1043, USA; Department of Psychology, The University of Texas at Austin, 108 E. Dean Keeton Stop A8000, Austin, TX 78712-1043, USA; Department of Psychology, The University of Texas at Austin, 108 E. Dean Keeton Stop A8000, Austin, TX 78712-1043, USA; Department of Psychology, The University of Texas at Austin, 108 E. Dean Keeton Stop A8000, Austin, TX 78712-1043, USA; Department of Psychology, The University of Texas at Austin, 108 E. Dean Keeton Stop A8000, Austin, TX 78712-1043, USA; Department of Psychology, The University of Texas at Austin, 108 E. Dean Keeton Stop A8000, Austin, TX 78712-1043, USA

**Keywords:** Key words: social dynamics, clusters, open field, population density

## Abstract

Environment is a determining factor that can facilitate or hinder social interactions. A precursor to meaningfully engaging with conspecifics is being exposed to opportunistic encounters with others. Increasing the number of individuals in a given space (thus increasing density) would, statistically speaking, increase the likelihood of accidental encounters. This might have consequences on the formation of social networks—an idea that has not reliably been explored. If true, we would expect that increasing density would lead to an increase in the number and the duration of ‘clusters’ of animals. Here, we examined whether varying the number of rats in an open field environment differentially affected their movement dynamics or their propensity to aggregate into clusters and, if so, whether such effects are dependent solely on statistical factors due to increases in density, the potential for actively-sought social interactions, or both. We found that the number of rats in an environment impacts ambulation speed, distance traveled, cluster formation and approaches, and that number and duration of clusters are highly dependent on the propensity for the rats to engage in social interactions.

## INTRODUCTIONS

There are both acute and chronic effects of increases in population density on animal behavior across a wide range of species, including humans [1–3]. Density dependence is indeed a highly prevalent and fundamental ecological process—it can impact access to resources, survival, and in turn, group dynamics [1–3]. But what might be the impact of a subtle and momentary difference in density? What impact might density have on basic movement dynamics of individual rats in an open environment?

A precursor to meaningfully engaging with conspecifics is to be exposed to opportunistic encounters with others. Arguably, increasing the density of individuals in a space is likely to increase the frequency of accidental social encounters and, as such, have consequences on the formation of social networks—an idea that has not reliably been explored. If true, we would expect that increasing density would lead to an increase in the number and duration of clusters, as well as the number of approaches rats make onto one-another. Opportunities for social interactions are advantageous as they could catalyze the formation of longer-term relationships. Affiliative bonding and kinship in humans and other animals alike can improve health outcomes ( [4–6]); yet, increases in population density can also increase stress [7, 8]. Studies have examined the effects of housing conditions (density, space) on a number of behavioral measures (e.g. [9]) in rats and mice, but no one has examined the effect of momentary density on movement dynamics in rodents.

What is the impact of physical density on the movement dynamics of rats? Here we examined whether group density influences how rats ambulate in an open environment. We further asked whether changes in group density increase the potential for incidental clustering, and whether the frequency of such encounters is solely dependent on the number of individuals in a given space, or depends on the individuals’ propensity for momentary interaction. We specifically examined if varying the number of rats in an open field environment would differentially affect their movement dynamics and their propensity to aggregate into clusters or engage in approach behavior toward others, and whether such effects would be dependent solely on differences in density, the potential for social interactions, or both.

## MATERIALS AND METHODS

All procedures were conducted in compliance with the National Institutes of Health Guide for the Care and Use of Experimental Animals and were approved by the Institutional Animal Care and Use Committee of the University of Texas at Austin.

### Overall experimental design

The overall experimental design simply involved placing a number of rats (details below) in an open field enclosure, and recording their movement. After approximately 4 minutes in the enclosure, the experimenter dropped food pellets (equal to the number of rats) in the middle of the arena.

Triads of male cage-mates were run in four conditions (one per day), in a counterbalanced design, as follows: with 12 other rats, 6 other rats, 3 other rats, or no other rats, yielding conditions with group sizes of 15, 9, 6, or 3, respectively. Rats were placed in an open field enclosure made of molded hard-plastic (Granger shipping container) measuring 41″ by 45″ by 18″ (W x L x H). The rats were allowed to interact freely for approximately 5 minutes until a food stimulus (banana pellets) was dropped in the center of the open field, and the rats were allowed to interact freely for approximately 5 minutes thereafter. We dropped as many banana pellets as the number of rats in the arena. Behavior was observed throughout and videotaped for later offline analysis. We analyzed the groups of 3, 6, 9, and 15 rats for speed, distance traveled, clustering, and approach behavior. To determine whether any clustering found effects were dependent on density, propensity for social interaction, or a combination of both, we ran bootstrapping analyses recreating resampled groups of 3, 6, 9, and 15 rats (see below).

### Housing

All rats were housed in triads in plexiglass shoebox cages in a reverse 12:12 day-light cycle, with lights off at 8 am. All experiments were run during the dark cycle under red light.

### Videotaping

All interactions were recorded from a fixed ceiling mounted camera (Sony, HandyCam) at 60 FPS, and saved for later offline analysis.

### DeepLabCut

**Figure 1:**
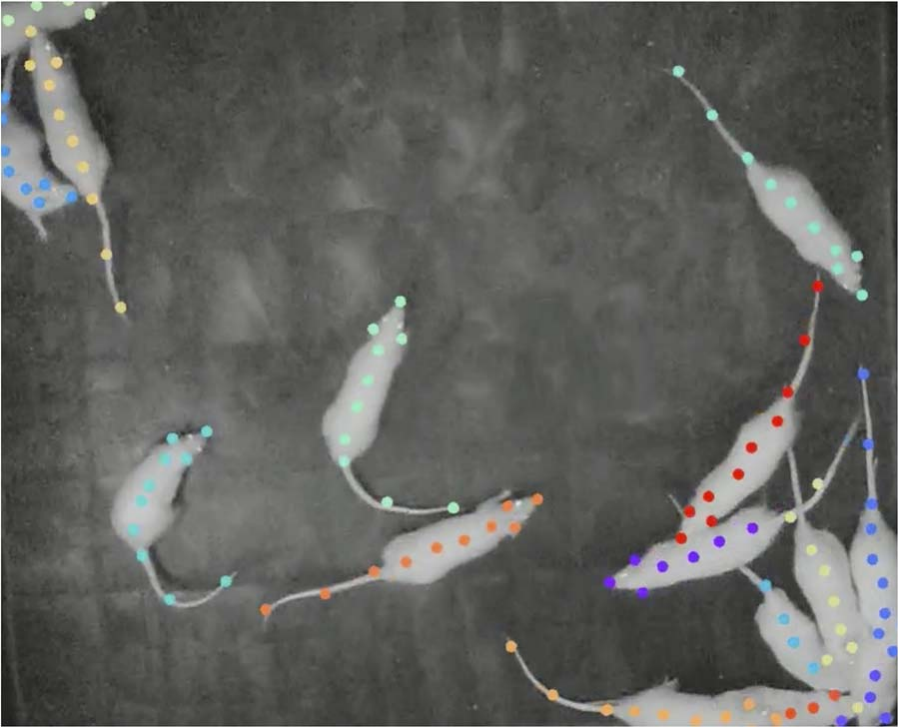
**Example frame of tracking for 15 rats.** An example video frame with the key points on the rats being tracked by DeepLabCut

To automate the tracking of individual rats in the arena, we used DeepLabCut (version 2.2b), an open-source positioning estimation software that employs deep residual neural networks to predict the location of previously labeled body parts on a trained dataset. A total of 60 trial videos with 20 frames from each video (900 total frames) were used to create training datasets for 4 different conditions (3, 6, 9, and 15 rats). For each, an experimenter manually labeled 10 individual body parts (nose, left and right ears, base of the neck, intermediary point, middle of the flank, second intermediary point, base of the tail, middle of the tail, and tip of the tail) of the rats (Fig. 1). The manually labeled frames were split into training (95%) and test sets (5%) and the networks were trained for 180 K–420 K iterations (3 and 9 rat models = 227 K each; 6 rat model = 420 K; 15 rat model = 180 K). The resulting body part positioning data were then exported as a csv file. DeepLabCut training was run on GPU powered Dell computers as well as remote GPU access through Google Collab.

### Raw data analyses

**Figure 2:**
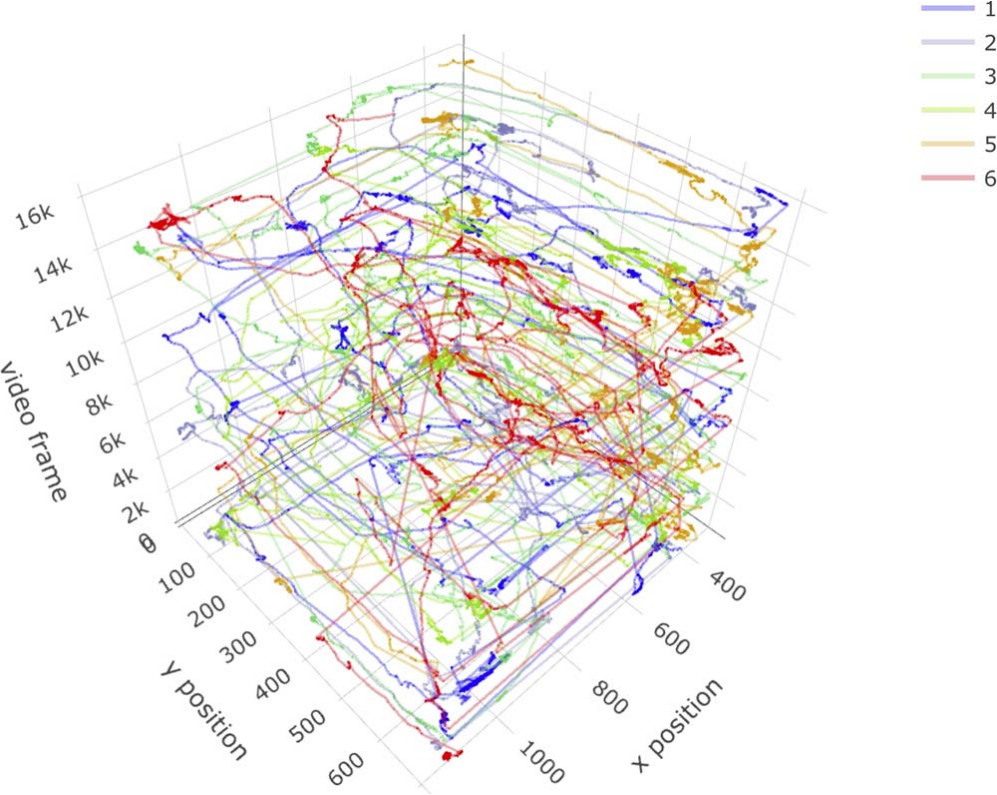
**Space–time paths for 6 rats.** An example of recovered space–time paths during a trial of the 6-rat condition. The x and y coordinates (in pixels) of the enclosure are shown on the floor of the plot, and time (in video frames) runs vertically. The plot shows ~4 minutes of data. These are the type of data that served as input to all subsequent analysis. Each colored line represents the path of one rat across space and time

All tracked videos (60, in total) were stored and visually inspected to ensure that tracking was suitable for analysis. The csv files generated from the DLC analysis were imported into R, and we wrote scripts to recreate each rat’s path in space across time for all 15 trials in each group size condition (See Fig. 2).

For each trial, we used the DBSCAN algorithm [8] to identify clusters of rats on a frame-by-frame basis (using the R package ‘fpc’). Here, a ‘cluster’ was defined as at least 3 rats with a minimum distance of 100 pixels (14.2 cm) from one another (the ‘eps’ parameter in the DBSCAN algorithm). After separating the clusters by size, we used run length encoding, to determine the duration or ‘lifetime’ of each cluster. Cluster lifetimes were calculated separately for clusters of different sizes. Thus, if a cluster of size 3 was joined by more rats, this larger cluster was considered a new cluster, and the calculated lifetime of the 3-rat cluster came to an end. And to be clear, the clusters were defined on the basis of the largest number of rats they contained. As such, a cluster of 6 rats was considered to be 1 cluster of 6 rats (not a cluster of 6, a cluster of 5, a cluster of 4, and 2 clusters of 3, for example).

We also calculated the momentary speed, total distance traveled, and the number of approaches for each rat in each condition. The speed was defined as pixel displacement per frame, and then transformed in cm/second. The total distance traveled was calculated as total pixel displacement, and then transformed in cm. The approach was quantified as any instance in which one rat’s snout positioning point came within 1 cm of any other point on another rat’s body.

### Bootstrapping

To determine whether any effects potentially observed between conditions were due to group density, propensity for social interaction, or an interaction between the two, we required a ‘baseline’ for the cluster sizes and lifetimes we would expect if all clusters formed by chance encounters alone, that is, clusters that were not driven by any social behavior on the part of the rats, be it approach or avoidance.

To do this, we randomly sampled the appropriate number of rat space–time paths from different trials in 2 different ways. We either sampled from the overall data pool, without regard for condition (overall bootstrapping), or within a given condition to recreate a bootstrap trial for that condition (within bootstrapping). In the former, for example, to create a bootstrapped trial for the 3-rat condition we randomly selected a trajectory from each of the 15 trials across conditions and ensured that no two paths were from the same run. Paths could thus come from one 3-rat run, one 6-rat run, and one 15-rat run. In the latter, to create a bootstrapped trial for the 9-rat condition, we randomly selected a trajectory from each of the 15 trials in the 9-rat condition, such that no two paths were sampled from the same actual trial. Thus, in either bootstrapping resampling, *any given trial consisted of paths that could not have been influenced by any of the other paths in that bootstrapped trial*. Any cluster formation in a bootstrapped trial resulted by chance, that is, not due to any social influence and with a probability determined solely by the rat density, and any global effects of rat movement due to that increase in density.

For every condition, we did 15 bootstrapped trials to mimic the real experiment. We did 2000 bootstrapped replications of the entire experiment, and subjected the data from each to the identical clustering analysis that was done for the real data. We performed the bootstrapping replications on Lonestar6, a supercomputer at the Texas Advanced Computing Center at the University of Texas at Austin.

### Data analyses

To examine whether there was an effect of number of rats on speed, distance traveled, cluster sizes, cluster duration, and number of approaches, we ran Factorial ANOVAs with Condition (number of rats) as a between-subject factor, and Food Drop as a within-subject factor in R. To examine whether there was a difference between our ‘real’ data vs/our bootstrapped samples, we plotted comparative histograms of the cluster sizes and durations. As we will see below, there was no need to formally compare the real distributions to the bootstrapped ‘chance’ distributions owing to the magnitude of the differences across all comparisons.

### Data availability

Raw data files (R code and csv files) are available in our Lab repository, housed at The University of Texas at Austin, https://doi.org/10.18738/T8/1VB8RR.

## RESULTS

For every outcome of interest, we tested whether there was an effect of condition (group size) on the experimental rats. We also compared the empirical distributions of cluster size and lifetimes to the corresponding bootstrapped distributions. The bootstrapped distributions represent a baseline or a ‘ground truth’ of how cluster sizes and lifetimes would be distributed if all encounters occurred by chance, with no influence of in-the-moment social factors. Any difference between the bootstrapped and real distributions, then, are due to the rats reacting to and interacting with other rats in the environment.

### Speed

**Figure 3:**
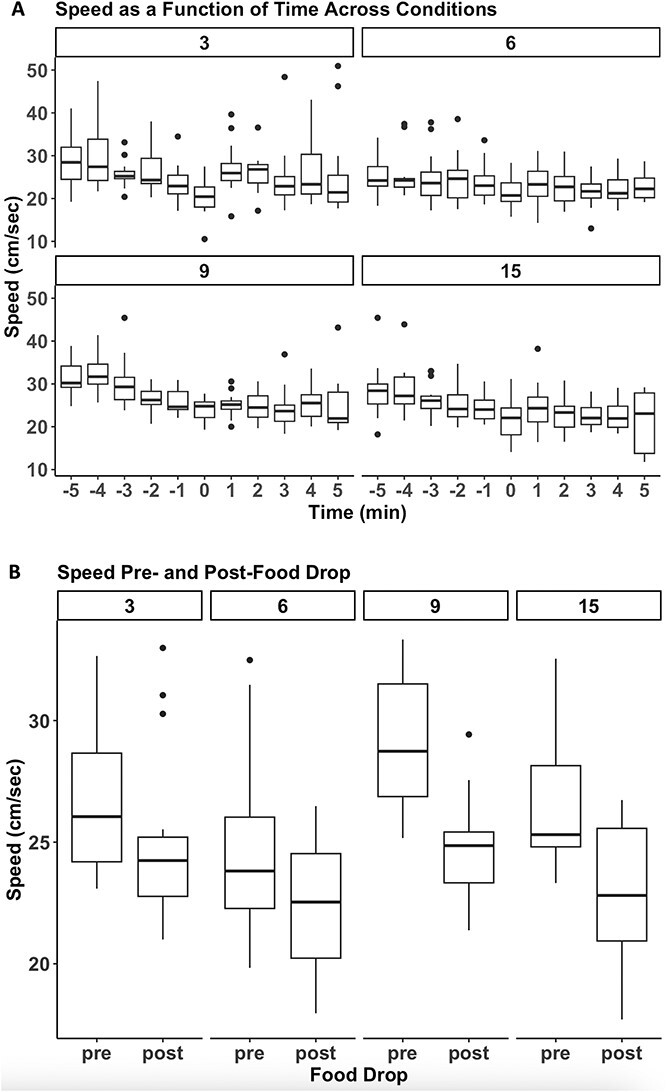
**Effects of number of rats on speed before and after food drop. A.** Boxplots of mean speed (in cm per sec) for each condition across time. The food pellets were dropped in the arena at time 0. **B.** Boxplots of mean speed (in cm per sec) estimates for the 4 conditions before (pre) and after (post) the food pellets were dropped. There was a statistically significant main effect of condition, and a statistically significant main effect of food drop. The rats’ speed decreased after the food compared to before, and there was a statistically significant difference in the rats’ speed between conditions. As evident from panel a, the main effect of food drop was predominantly due to a habituation effect across time for all conditions

As shown in Fig. 3, Panel A, the rats’ speed showed habituation across time spent in the arena, and there were overall differences between the groups. There was a significant main effect of Time, F(10, 440) = 10.787, p < 0.0001, and a significant main effect of condition, F(3, 44) = 4.101, p = 0.001. As shown in Fig. 3, Panel B, there was a significant main effect of condition (number of rats), F(3, 56) = 4.967, p = 0.0004, and a significant main effect of Food drop, F(1, 56) = 57.285, p = 3.97^e-10^ on speed, but no significant (though a trend toward) interaction, F(3, 56) = 2.196, p = 0.09. Follow up tests revealed that rats in the 9-rat condition ambulated faster than any of the other groups (p < 0.05, all contrasts). Overall, the rats’ speed decreased after the food pellets were dropped in the arena compared to before. Follow-up planned comparisons with Bonferroni correction revealed that there was a significant difference pre- and post-food drop in the 9- and 15-rat conditions (p < 0.005, each comparison), and a trend for the 6-rat condition (p = 0.052).

### Distance travelled

**Figure 4:**
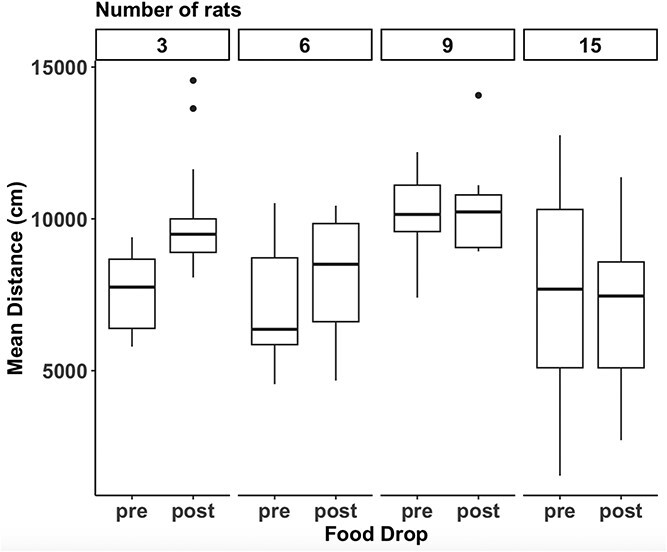
**Effects of number of rats on distance traveled before and after food drop.** Boxplots of total distance traveled by the rats in the 4 conditions before and after food pellets were introduced into the enclosure. There were statistically significant main effects of food drop and condition, and a statistically significant food drop by condition interaction. Specifically for the 3-rat condition, the rats moved a significantly greater distance after the food was dropped in the arena

As shown in Fig. 4, there was a significant main effect of condition, F(3, 56) = 8.203, p < 0.001, a significant main effect of Food Drop, F(1, 56) = 6.76, p = 0.012, and a Condition by Food Drop interaction, F(3, 56) = 6.021, p = 0.001. Overall, the rats increased the distance they traveled once the food pellets were dropped in the arena, and the distance traveled was different between the groups. Follow up tests revealed that all significant effects were predominantly driven by the 3-rat condition, where the distance traveled increased significantly after the food pellets were dropped, p < 0.005. None of the other conditions showed significant differences. The differential effect on the 3-rat condition was at the source of the interaction.

**Figure 5:**
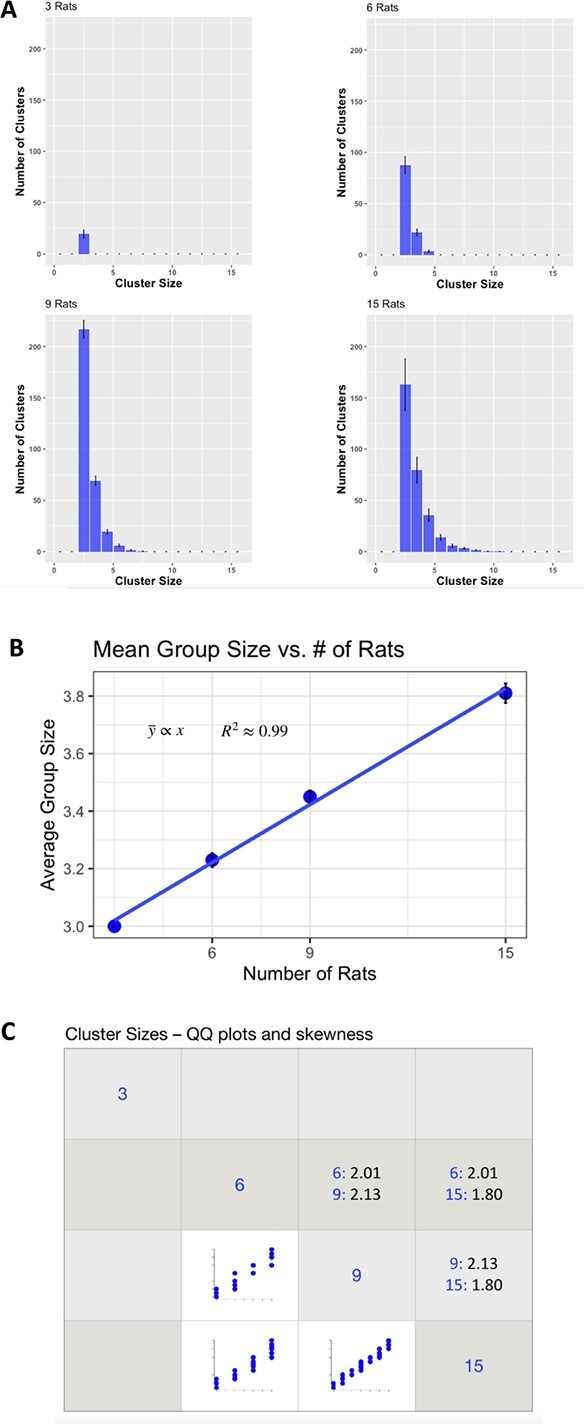
**Relationship between condition and cluster size. A.** Histograms of the cluster sizes for the all conditions. The most common cluster size was 3 for all conditions, but the distributions flattened, and the right tails grew as the number of rats increased. **B.** Regression of the relationship between cluster size as a function of condition (number of rats). There was a strong statistically significant relationship; number of rats explained 99% of the variance in cluster size. As the number of rats increased, the mean cluster size increased. **C.** A matrix showing pairwise comparisons between the cluster size distributions. The cells in the lower triangle show the pairwise quantile-quantile (QQ) plots between condition and cluster size. These indicate that the shape of the distributions is invariant across group size. Consistent with visual inspection of the distribution histograms, all the distributions are highly skewed (a Gaussian having a skewness of 0) The cells in the upper triangle show the actual skewness value, showing that the distributions are positively skewed.

### Clustering across conditions

We examined the rats’ propensity to assemble into clusters, as a function of number-of-rats condition. Panel A of Fig. 5 shows the distributions of cluster sizes formed for each condition. In our raw data samples, visual examination and our analyses revealed differences in clustering between our conditions. Effectively, as the number of rats increased, we observed an increase in the total number of clusters (See Fig. 5). A between-groups comparison of number of 3-rat clusters formed by the different conditions was significant, F(3, 56) = 35.47, p < 0.0001 showing that overall, as the number of rats increased in the enclosure, there was an increase in the number of 3-rat clusters formed (the effect plateaued with the 9-rat condition). The most common cluster size was 3 for all conditions, but the distributions flattened, and the right tails grew as the number of rats increased, simply highlighting that whereas 15 rats can assemble into clusters of many different sizes, 3 rats can only form clusters of 3 at the most. As shown in Panel B of Fig. 5, a regression of the relationship between cluster size as a function of number of rats in the enclosure revealed a strong linear relationship; number of rats explained 99% of the variance in cluster size, p < 0.005. As the number of rats increased, the mean cluster size increased. We show quantile-quantile (QQ) plots to compare the shape of the different distributions as a function of condition and cluster size. Panel C of Fig. 5 shows the pairwise QQ plots between condition and cluster size (cells in the lower triangle), which indicate that the shape of the distributions is invariant across group size. The cells in the upper triangle show the actual skewness value, showing that the distributions are positively skewed. The condition distributions for 6, 9, and 15 rats showed skewness of 2.01, 2.13, and 1.80, respectively.

**Figure 6:**
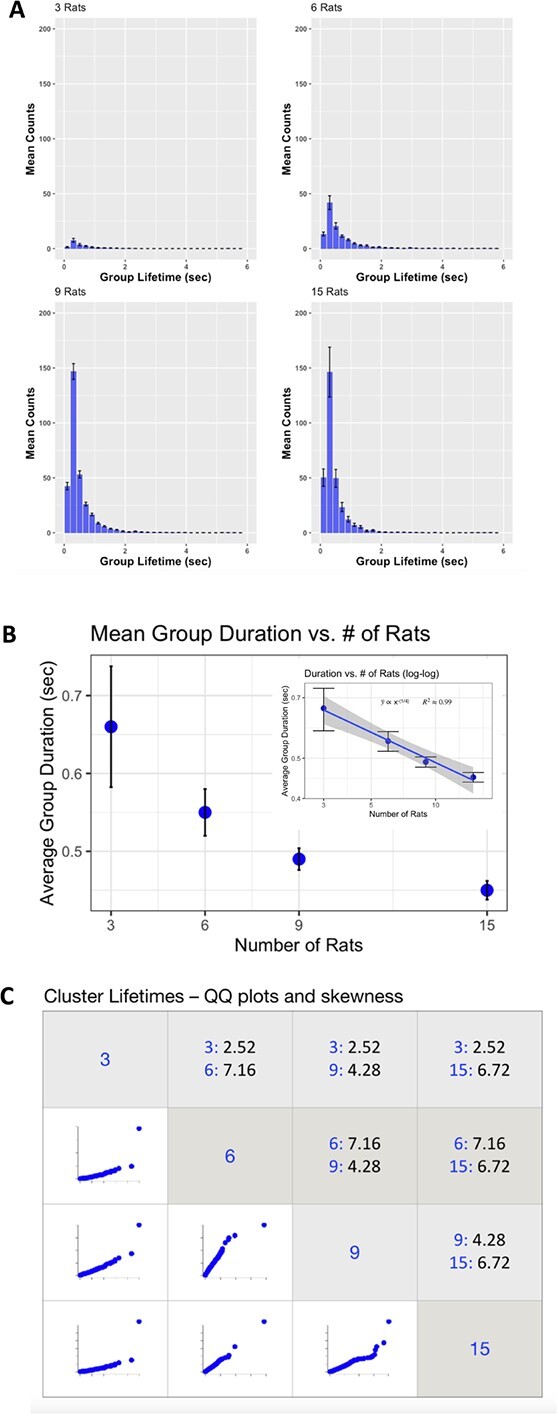
**Relationship between condition and cluster duration.** A. Histograms of cluster duration for all conditions. The most common cluster durations were between 200 and 400 msec, but the tail of all the distributions extends passed 2 seconds. B. Relationship between condition (number of rats) and overall cluster duration, showing that as the number of rats in the arena increased, the cluster duration decreased exponentially. C. A matrix showing pairwise comparisons between the cluster duration distributions. The cells in the lower triangle show the pairwise quantile-quantile (QQ) plots. The QQ plots indicate that the distributions are generally the same shape except for the extreme right tails. The cells in the upper quadrant of the matrix shows the actual skewness values. Consistent with visual inspection of the distribution histograms, all the distributions are highly skewed (a Gaussian having a skewness of 0). Any large differences in numerical skewness are generally due to only the upper two quantiles.

We also examined the relationship between number-of-rats condition and cluster duration. In Fig. 6, panel A, we show the histograms of cluster duration for the 3, 6, 9, and 15 rat conditions. The most common cluster durations were between 200 and 400 msec, but the tail of all the distributions extends past 2 seconds. In Fig. 6 panel B, we show the relationship between condition (number of rats) and overall cluster duration—as the number of rats in the arena increased, the cluster duration decreased in a linear log–log relationship shown in the inlet of Panel B. Number of rats accounts for 99% of the variance in cluster duration. In Panel C, we show the matrix of pairwise comparisons between the cluster duration distributions. The cells in the lower triangle show the pairwise quantile-quantile (QQ) plots, which indicate that the distributions are generally the same shape except for the right tails. The cells in the upper quadrant of the matrix show the actual skewness values. Consistent with visual inspection of the distribution histograms, all the distributions are highly skewed (a Gaussian having a skewness of 0), with the condition distributions for 3, 6, 9, and 15 rats showing skewness of 2.52, 7.16, 4.28, and 6.71, respectively. Any large differences in numerical skewness are generally due to only the upper two quantiles.

**Figure 7:**
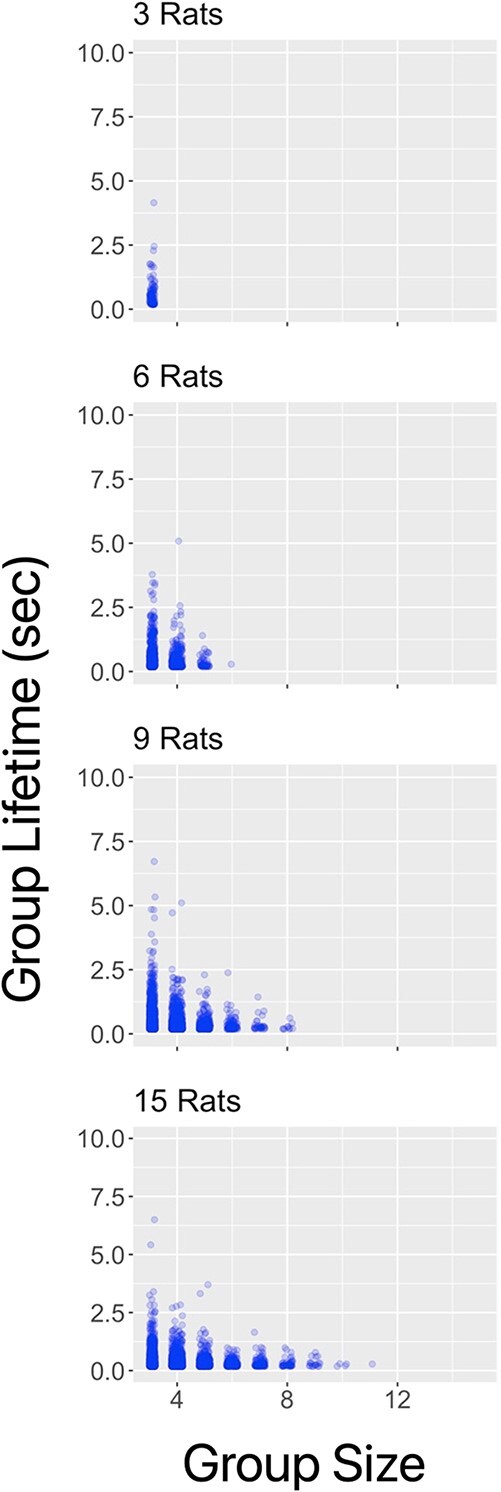
**Distribution of cluster duration as a function of cluster size for the 4 conditions (number of rats).** The data points are jittered on the x-axis for clarity. While most clusters are small and short-lived, smaller clusters are related to longer lifetimes. For example, clusters of size 3 often last longer than 2 seconds, whereas clusters of size 6 almost never do. Note that this figure combines and replots the data from the first panels of the previous 2 figures, but this plot shows the tails of the duration distributions more clearly and highlights the relationship between cluster size and duration across conditions

In Fig. 7 we illustrate the individual datapoint distributions of cluster lifetime across conditions, further highlighing the effect of cluster duration as a function of cluster size—the smaller clusters (3 rats) lasted longer than the larger ones, and the effect was consistent across conditions. The distribution of cluster sizes was different between conditions, owing in part to the different cluster size possibilities between the conditions (e.g. whereas a group of 6 rats can aggregate into groups of 3, 4, 5, or 6 rats, a group of 15 rats can do so into formations of 3, 4, 5, 6, 7, 8, 9, 10, 11, 12, 13, 14, or 15). Still, the distributions revealed few occurrences of clusters of 6 or more rats in the 9-rat condition, or of 9 or more rats in the 15-rat conditions (See Fig. 6). Across all, the most stable number, that is the cluster size that was most enduring, was 3 (see Fig. 7). Interestingly, this was especially true of the 3-rat condition, which featured a heavier right tail than the other conditions (See Fig. 6, Panel A). In sum, while most clusters are small and short-lived, smaller clusters are related to longer lifetimes.

### Real vs. bootstrapped experiments

**Figure 8:**
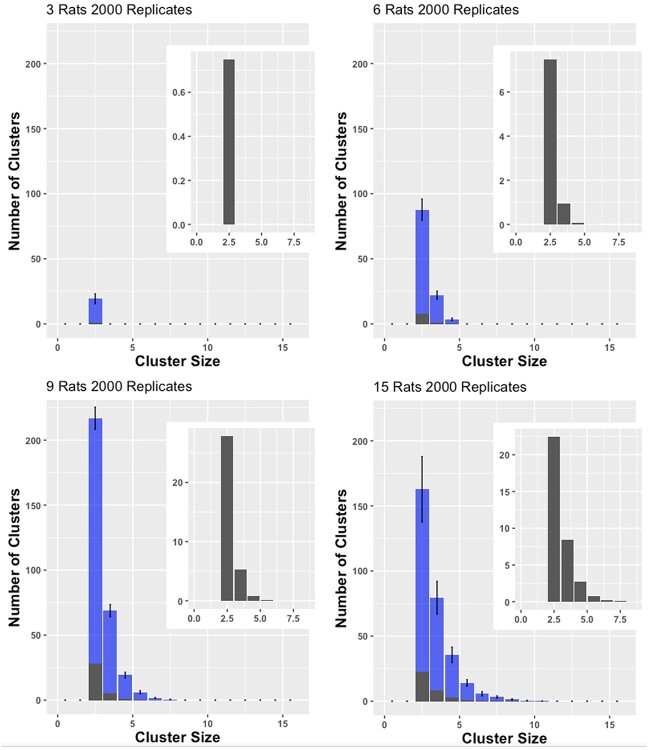
**Histograms of the distributions of cluster sizes from the actual experiment (error bars are +/− standard error of the mean) and the within bootstrapped replications (overlay, also shown in inset).** The distributions are mean counts. For all conditions, there were vastly more clusters formed by the ‘real’ rats than were formed by their bootstrapped counterparts , which were unaware of the behavior of their virtual enclosure-mates

**Figure 9:**
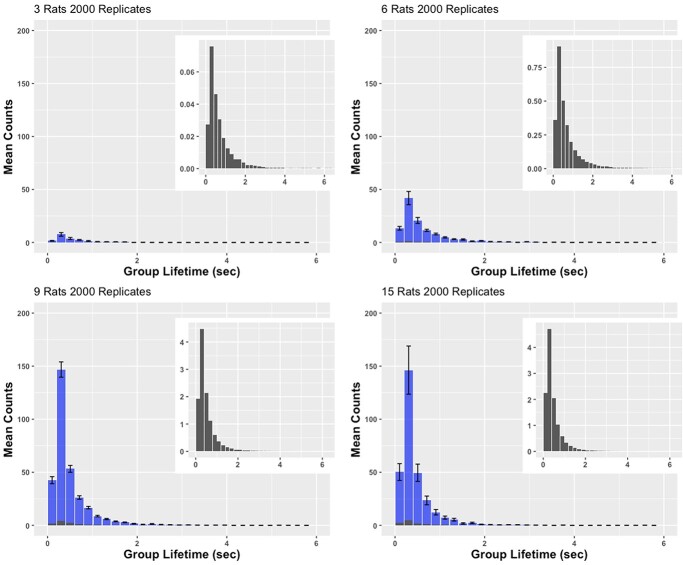
**Histograms of the distributions of cluster durations from the actual experiment (error bars are +/− standard error of the mean) and the within bootstrapped replications (overlay, also shown in inset).** The distributions are mean counts. For all conditions, more clusters (by at least a factor of 3 for most durations) were formed by the real rats than by their virtual bootstrapped counterparts. The real rats always clustered more in groups with modal lifetimes of around 200 to 400 msec

The difference between the clustering in the real experiments and clustering in the bootstrapped experiments was striking, showing that the rats do have a propensity to cluster in an environment in which they can physically interact in real-time far over and above what would be predicted by chance encounters due to rat density. Figure 8 shows the empirically obtained distribution of cluster sizes for the four conditions (blue); overlaid are the within bootstrapped distributions across 2000 bootstrap replicates. Clearly, in all four conditions, the rats in the actual experiment form a lot more clusters (by an order of magnitude), than the bootstrapped rats, which do not possess the ability to react to or interact with other rats in real-time. As the number of rats increases across conditions, so does the probability of chance encounters. For the number of 3-rat clusters formed (the only cluster size that is achievable by all conditions), we show a main effect of condition for the Real, overall bootstrapping, and within bootstrapping samples (F(3, 56) = 35.47, p < 0.0001; F(3, 56) = 164.49, p < 0.0001; F(3, 56) = 184.11, p < 0.0001, respectively). The increase in number of 3-rat clusters plateaued when there were 9 rats in the arena for the Real as well as bootstrapped samples, actually showing the reverse relationship between the 9- and 15-rat conditions (15-rat <9-rat >6-rat >3-rat; p < 0.05, all contrasts). Still, despite a statistically significant difference across conditions for each subsample, the effect paled in comparison to the difference between the Real and Bootstrapped samples (see Figs. 8 and 9, and Supplementary Figs. 1 and 2).

Figure 9 shows the distributions of actual cluster lifetimes (blue) with the distribution of lifetimes from the bootstrapped experiments superimposed (2000 replicates). When rats are in the environment (Real data, in blue), they form many more clusters across all conditions, at all lifetimes. In other words, real rats (shown in blue) cluster more and for longer than would be expected from chance encounters (estimated by the bootstrapped samples, in black).

### Approach behavior

**Figure 10:**
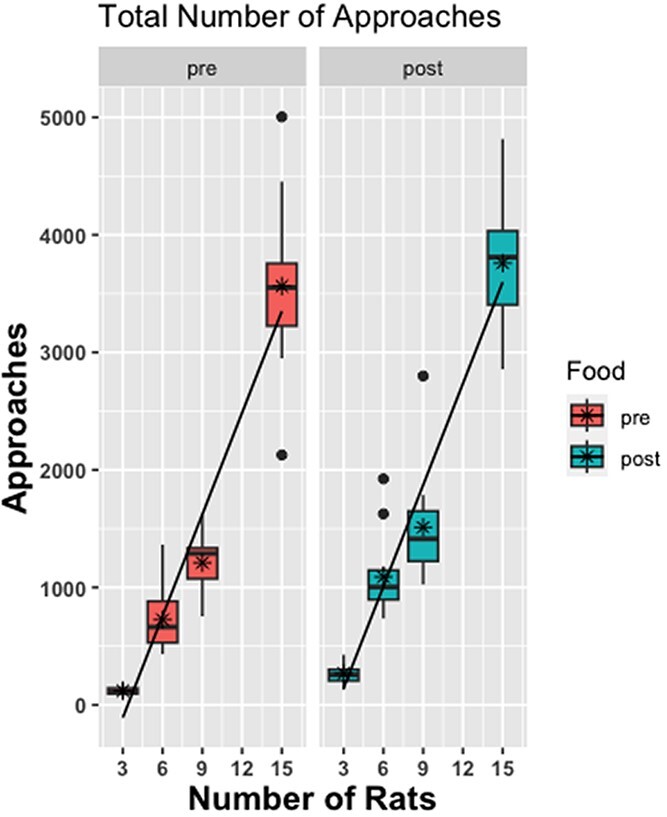
**Boxplots of the total number of approaches for the 4 group size conditions pre and post food drop.** There was a greater number of total approaches (across groups) as group size increased

**Figure 11:**
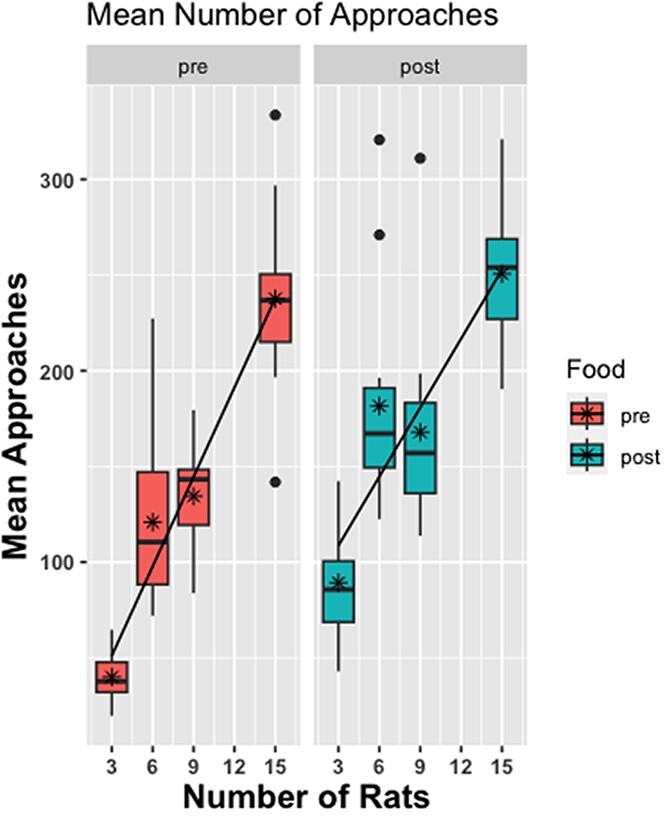
**Boxplots of the mean number of approaches for the 4 group size conditions pre and post food drop.** There was a greater number of mean approaches (across groups) as group size increased. As the group size increased, there was an increased number of approaches for each rat on average

There was a significant main effect of condition on the total number of conspecific approaches, which increased as the number of rats increased (see Fig. 10), F(3, 56) = 343.457, p < 0.0001 (where 15-rat >9-rat >6-rat >3-rat, p < 0.05, all contrasts). Figure 11 shows the same effect normalized to the number of rats (mean approaches). As shown in Figs. 10 and 11, there was also a significant main effect of Food Drop, where the number of approaches increased after the pellets were dropped in the arena relative to before, F(1, 56) = 19.481, p < 0.0001. There was no condition by food drop interaction.

## DISCUSSION

Rats, like humans, are social animals and are affected by their surroundings. Many studies have examined the impact of extreme social manipulations on behavior (e.g. social isolation; overcrowding) in rats [11]. We were interested in a more subtle approach. As premise for the present experiment, we wondered what might be the ontogeny of social interactions, and whether accidental encounters with conspecifics might serve as a basis for affiliative kinship in rats. We hypothesized at onset that given the opportunity to interact together in an open environment, rats would engage in approaching others, and would aggregate into clusters. We further hypothesized that increasing the number of individuals in a given space would affect their locomotion, as well as lead to a commensurate increase in the likelihood of chance encounters, which in turn would positively impact the propensity to aggregate. We specifically tested whether varying the number of rats in an open field environment differentially affects their movement dynamics or their propensity to aggregate into clusters or engage in approach behavior toward others, and whether such effects are dependent solely on the statistics of differences in density, potential for social interactions, or both. We found that rat density influences movement dynamics, propensity to aggregate into clusters, as well as approach behavior, and that these effects are influenced by the potential for social interactions, not just the statistical increase in the chance of random cluster formation.

### Rat density influences movement dynamics in an open environment

Our findings suggest that locomotion was faster across the groups of rats at the beginning of the session but settled at a stable value after a few minutes. Interestingly, access to a food source had no impact on locomotion speed—any pre/post food effect could be explained by habituation. When the banana pellets were dropped into the environment mid-way through the session, however, rats from the smallest density condition (3-rat) traveled a greater distance than they had for the first half of the session. While access to food is clearly important for rats in the wild, the rats in our experiment were not food deprived, so the stakes of deciding to forego traveling to procure the food item were not as high. It may thus be somewhat surprising that a subset of the rats actually increased travel distance to obtain the food, and arguably did so when there was less competition for the food (in the 3-rat condition). Furthermore, rats generally showcase neophobia to novel food sources. Since they were not a priori habituated to the banana pellets before receiving them in the arena, it is possible that a few rats that were perhaps bolder on the shyness-boldness [12] scale decided to try the pellet, and once they did, other rats followed suit. The fact that the effect occurred in the 3-rat condition might suggest that in the smaller group, rats pay closer attention to other rats as they learn from their surroundings. In future studies, we plan to evaluate the rats’ hierarchical standing within their triad before testing their group behavior to determine if dominance might influence group dynamics beyond their triadic formation.

We should note that all our rats were housed in triads, and we did not examine the effects of chronic density increases on behavior. Others previously found that a modest increase in rat numbers in the home cage led to increases in locomotion speed in an open field environment when rats were tested individually [13]. This effect was found to be dependent on developmental age, with juveniles being the most affected, and suggestive of an effect of homecage dominance dynamics on stress levels [13].

Whereas many groups have examined the effects of housing conditions (density, space, enrichment) on performance in a number of behavioral tasks (e.g. [9]) in rats and mice, no one previously looked at the effect of individual density on movement dynamics in rodents. In humans, there is a reported relationship between population density and behavior. For example, as the number of individuals in a population increases, there is a commensurate increase in walking velocity [14]. Interestingly, for every 1% increase in population density, there is a 0.1% increase in donations to political campaigns [15]. Such effects, though, appear driven not solely on the basis of increased density, but characteristics of the individuals in the population, including their affiliative kinship. The effects of population density on locomotion velocity also appears dependent on factors beyond density itself—the number of individuals that must *interact* is a variable that significantly impacts behavior. Furthermore, increasing the number of individuals in space also comes with increased levels of social stimulation and inevitable social interaction, along with real or perceived violation of personal space ([7, 14]). When there are no other people around, there is an increase in physiological arousal, as measured by skin conductance responding, with increases in walking speed; however, the reverse effect is observed when the density increases to the point of having to slow down due to the presence of other people. In such scenarios, physiological arousal increases at lower speeds, possibly indicative of increased stress [7]. Thus, variations in walking speed alone do not predict arousal—the effect is situationally dependent. Along similar lines, the aforementioned increase in political donations is most prevalent amongst democrats, and one of the most influential factors is whether a person close to the donor has also chosen to donate [15]. Taken together, there seems to be an effect of group density on behavior, though this effect may be moderated by social interactions. In a broad sense, this is in-line with our findings—we observed an effect of density on clustering dynamics, but this effect is dependent on the propensity to engage in social interactions with other conspecifics.

### Rat density influences propensity to assemble

As the number of rats increased, we observed an increase in the total number of clusters over and above what would be predicted by chance. We also observed an effect of cluster duration as a function of cluster size—the smaller clusters (3 rats) lasted longer than the larger ones, and the effect was consistent across conditions. These findings support our a priori hypothesis that increasing density would lead to an increase in the propensity to aggregate. Further consistent with our hypothesis, our analyses revealed that the total number of conspecific approaches generally increased as the number of rats increased. This is perhaps not surprising, given that a larger number of rats yields a greater potential for encounters.

### The potential for social interaction influences density-dependent movement dynamics and propensity to aggregate in rats

Our comparison of the real data to those from the bootstrapped samples revealed that there were far fewer clusters formed when there was no potential for the rats to engage with one another in real-time. It is thus clear that rats modify their space–time paths based on other rats in the environment—they *purposely* cluster with other rats. To be perfectly clear, the space–time paths for a given rat in a bootstrapped trial have also been modified by other rats, but *not by the rat paths with which they have been combined in a bootstrapped trial*. One can think of it this way: each rat in a bootstrapped trial is reacting to other rats, but they are reacting to ‘ghost rats’ not present on the current trail, and each rat in the current trial is reacting to a different set of ghost rats. Any clustering in a bootstrapped trial must therefore be due to chance meetings of paths. These results support the notion that group aggregates form not only because of density, but density when rats can meaningfully interact (and disproportionately-so when they can meaningfully interact).

### Conclusion

Our study examined the effects of momentary increases in rat density on movement dynamics and propensity to aggregate into clusters and engage in approach behavior. Our results suggest that even brief situational increases in individual density in an environment affect rats’ movements, clustering, and approach behavior — factors that could in turn have an impact on health and well-being. For decades, many animals have experienced momentary density changes from displacement owing to human (e.g. construction) and natural (e.g. wildfires) factors. Climate change’s impact on the world has set in motion increased uncertainty with respect to living conditions and livable spaces on earth. It is likely that many humans and other animals will incur, on a global scale, sudden and pronounced displacement—this fact carries the likelihood that we will also face increases in density. Developing a comprehensive view of the impact of density changes ushered in will be essential through this transition.

## STUDY FUNDING and APC FUNDING

The work presented therein was funded by grants (2141102 and 1748911) from The National Science Foundation (NSF).

## AUTHORS’ CONTRIBUTIONS

Marie Monfils (Conceptualization-Lead, Data curation [Equal], Formal analysis [Lead], Funding acquisition [Lead], Investigation-Lead, Methodology [Lead], Project administration [Lead], Resources [Lead], Supervision [Lead], Validation [Lead], Visualization [Equal], Writing – original draft [Lead], Writing – review & editing [Equal]), Michael Pasala (Formal analysis [Equal], Methodology [Equal], Visualization [Equal], Writing – review & editing [Equal]), Cassidy Malone (Data curation [Equal], Methodology [Equal], Writing – review & editing [Equal]), Laura Agee (Investigation [Equal], Writing – review & editing [Equal]), Rheall Roquet (Investigation [Equal], Writing – review & editing [Equal]) and Lawrence Cormack (Data curation [Equal], Formal analysis [Lead], Validation [Lead], Visualization [Equal], Writing – review & editing [Equal]).

## CONFLICT OF INTEREST STATEMENT

None declared.

## Supplementary Material

Web_Material_kvae005
