# Supplementary material for: Effects of group size on movement patterns and clustering dynamics in rats
Source: Oxf Open Neurosci. 2024 Apr 2;3:kvae005. doi: 10.1093/oons/kvae005 (PMC11019387; doi:10.1093/oons/kvae005)
Supplement: Web_Material_kvae005 [file web_material_kvae005.zip › Supplementary Information.docx]

Supplementary Information


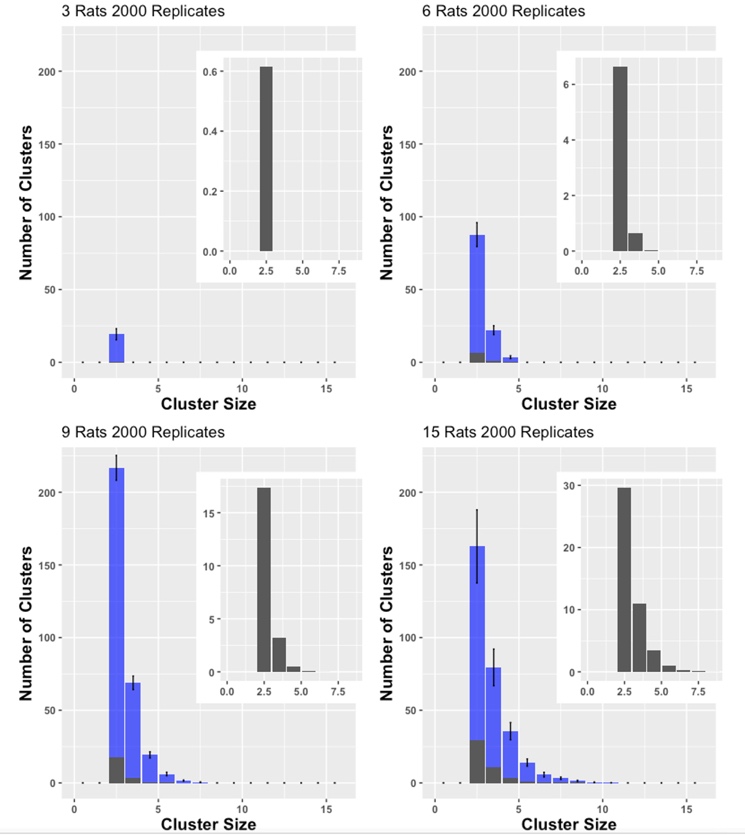


**Supplementary Figure 1. Histograms of the distributions of cluster sizes from the actual experiment (blue; error bars are +/- standard error of the mean) and the overall bootstrapped replications (black).** The distributions are mean counts. For all conditions, there were vastly more clusters formed by the “real” (blue) rats than were formed by their bootstrapped counterparts (black), which were unaware of the behavior of their virtual enclosure-mates.


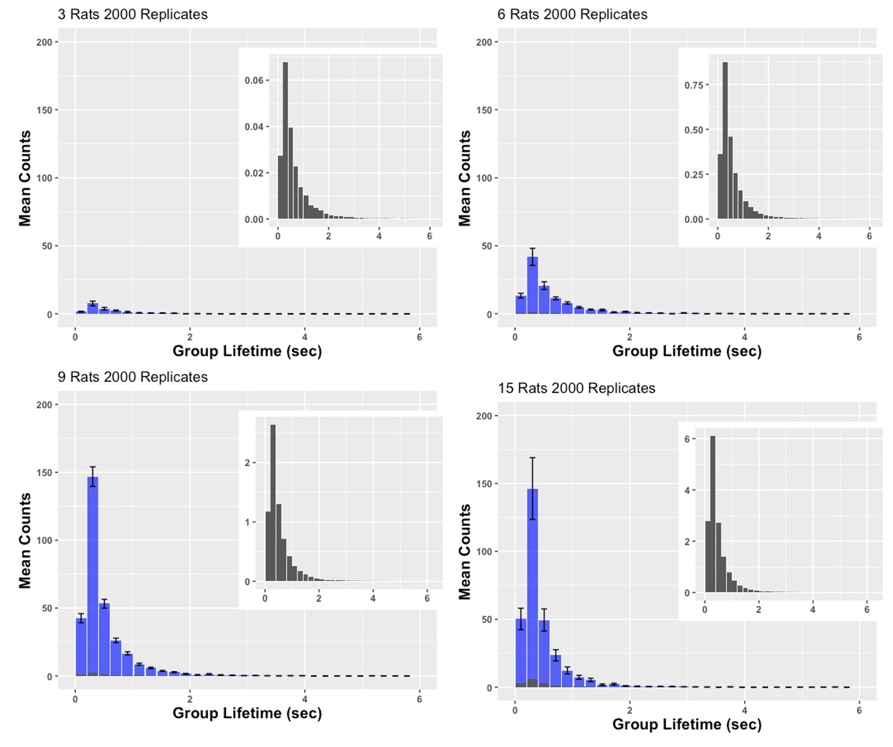


**Supplementary Figure 2. Histograms of the distributions of cluster durations from the actual experiment (blue; error bars are +/- standard error of the mean) and the overall bootstrapped replications (black).** The distributions are mean counts. For all conditions, more clusters (by at least a factor of 3 for most durations) were formed by the real rats than by their virtual bootstrapped counterparts. The real rats always clustered more in groups with modal lifetimes of around 200 to 400 msec.
