# Supplementary material for: Effects of group size on movement patterns and clustering dynamics in rats
Source: Oxf Open Neurosci. 2024 Apr 2;3:kvae005. doi: 10.1093/oons/kvae005 (PMC11019387; doi:10.1093/oons/kvae005)
Supplement: Web_Material_kvae005 [file web_material_kvae005.zip › Reviewers comments - Supplementary Material - OXFNSC-2023-017 .docx]

**REVIEWER COMMENTS**

**OXFNSC-2023-017 - Effects of group size on movement patterns and clustering dynamics in rats**

**Original submission**

**Reviewer 1**

Comments to the Author

Authors use an interesting design to test locomotion in varying social grouping scenarios. Rats are put into an open field and locomotor measures (distance, approach, etc) were quantified in the presence of 3 to 15 other conspecifics to investigate movement dynamics with social stimuli. This paper is interesting but lacks some details that are critical for some of the conclusions made. I’ve detailed them below.

Major

• In the introduction, it may be helpful to keep the first paragraph to relevant information in the paper. For example, authors are not looking at long-term social dynamics with changes in group living density, so information like infanticide and cannibalism are not relevant. Instead, literature on hierarchies, novel social interactions, social satiation, etc would be more helpful to set up the present results.

• In the bootstrapping section, it would be helpful to define what a trajectory is defined as. It’s unclear if this refers to tracking a single rat for the entire session or until a goal is met (e.g. reached a social contact, food pellet). If until a goal is met, when was the start and end of a trajectory defined?

• How exactly were clusters defined? Was a group of 6 rats considered 6 for the entire social bout or was it first considered a cluster of 3 until other rats joined? It may be helpful to look at locomotor dynamics between rats that ‘initially’ formed clusters and rats that ‘joined’ clusters.

• Please report full statistics in the results section for each graph/figure

• Authors should account for changes in social vs nonsocial locomotion. For example, chasing/following behaviors vs approach vs movement that does not result in physical contact or proximity to another group

• Authors state that they account for social familiarity, but it’s difficult to interpret where and how this is accounted for. For each grouped scenario (3, 6, 9. 15 rats), were all rats novel to one another, cagemates, or had prior interactions? If familiar, how did interaction change between those conspecifics with larger group sizes? I think I understand that authors state this was based on a ‘baseline’ session to assess familiarity, but did authors log interaction time between conspecifics as an index for familiarity? This influences subsequent behaviors with other conspecifics, so it would be helpful to be as clear as possible and quantify novel vs familiar conspecific interaction times or timing until ‘familiarity’ is achieved based on baseline performance. This should be done outside of the bootstrapping, as ‘real’ and ‘bootstrapped’ experiments differed.

• For the food pellet condition, it is unclear what traveling a great distance means. Do rats travel a greater distance for the pellet, does the food pellet introduce enough novelty that rats increase exploratory movement? How is clustering in larger group conditions accounted for in distance measures here? If a single food pellet went to a single rat, what were the locomotor characteristics of this particular rat during the session before and after? Did this rat consistently receive the pellet across sessions?

• Instead of total clusters, it may be more helpful to examine distance measures between when clusters were formed and time and how clusters influence approach for other conspecifics. This would provide an additional level of detail regarding propensity to socially engage or locomotion for social opportunity.

Minor

• In the methods section, authors may fill in a number for the ‘XX’ in the paragraph discussing the DBSCAN algorithm

• In other ‘words’ vs works in the Bootstrapping section

**Reviewer 2**

Comments to the Author

Authors examined how the number of rats affect movement dynamics and clustering, and whether such effects depend on population density or seeking of social interaction, or both. Behaviors were recorded on camera and analyzed using DeepLabCut to assess clustering, speed, and distance. These effects were analyzed in terms of density, familiarity or both. Overall, these results represent an important line of investigation into the ethological factors of motivated behavior in groups. However, there is a need to include more statistics in the manuscript before one can judge the contribution fairly and rigorously.

My major critique is that there is little statistical reporting of any of the Figures in the Results section. Authors state they performed Kolmogorov-Smirnov tests but I could not find these in the Results, just in the Figures. As presented in the Figures, there is no clear description. Authors need to include a text description of the results of the statistical tests. Even in the case that the results are descriptive and do not test specific hypotheses, i.e. “the total number of conspecific approaches generally increased as the number of rats increased” authors should provide means and SEM and/or tests for these in the Results section.

Authors need to improve/increase the size and legibility of axes labels and standardize the look of them across the Figures.

It is unclear how the authors separated the effects observed between conditions as due to either group density or pre-established social familiarity, particularly the latter. Please include more detail. Was housing (i.e., being cage mates) entered into the analysis? If not, authors should consider conducting such an analysis perhaps with regression and/or GLM with familiarity (cage mate or not) as a formal predictor of movement.

Minor

Please delete (line 12, page 5): though we are confident that the results won’t change, but our precision will increase of course.

**Decision letter**

28-Dec-2023

Dear Dr. Monfils,

Manuscript ID OXFNSC-2023-017 entitled "Effects of group size on movement patterns and clustering dynamics in rats" which you submitted to the Oxford Open Neuroscience, has been reviewed. The comments of the reviewers are included at the bottom of this letter.

Please note that this journal operates with transparent peer review. This means that if your submission is eventually accepted for publication, the full peer review history of your article will publish online alongside your article. This includes reviewer comments, editor decision letters, and your author responses.

The reviewers suggest some major revisions to your manuscript. Therefore, I invite you to respond to the reviewers' comments and revise your manuscript within the next 60 days from today date.

To revise your manuscript, log into https://mc.manuscriptcentral.com/oxfnsc and enter your Author Centre, where you will find your manuscript title listed under "Manuscripts with Decisions." Under "Actions," click on "Create a Revision." Your manuscript number has been appended to denote a revision.

You may also click the below link to start the revision process (or continue the process if you have already started your revision) for your manuscript. If you use the below link you will not be required to login to ScholarOne Manuscripts.

*** PLEASE NOTE: This is a two-step process. After clicking on the link, you will be directed to a webpage to confirm. ***

https://mc.manuscriptcentral.com/oxfnsc?URL_MASK=7a99f598a1474ef3990e0701e1181fa8

You will be unable to make your revisions on the originally submitted version of the manuscript. Instead, revise your manuscript using a word processing program and save it on your computer. Please also highlight the changes to your manuscript within the document by using the track changes mode in MS Word or by using bold or colored text.

Once the revised manuscript is prepared, you can upload it and submit it through your Author Centre.

When submitting your revised manuscript, you will be able to respond to the comments made by the reviewers in the space provided. You can use this space to document any changes you make to the original manuscript. In order to expedite the processing of the revised manuscript, please be as specific as possible in your response to the reviewers.

IMPORTANT: Your original files are available to you when you upload your revised manuscript. Please delete any redundant files before completing the submission.

Because we are trying to facilitate timely publication of manuscripts submitted to the Oxford Open Neuroscience, your revised manuscript should be uploaded within the next 60 days from today date. If it is not possible for you to submit your revision in a reasonable amount of time, we may have to consider your paper as a new submission.

Once again, thank you for submitting your manuscript to the Oxford Open Neuroscience and I look forward to receiving your revision.

Sincerely,

Dr. Alicia Izquierdo

Senior Editor, Oxford Open Neuroscience

aizquie@psych.ucla.edu

Reviewer: 1

Comments to the Author

Authors use an interesting design to test locomotion in varying social grouping scenarios. Rats are put into an open field and locomotor measures (distance, approach, etc) were quantified in the presence of 3 to 15 other conspecifics to investigate movement dynamics with social stimuli. This paper is interesting but lacks some details that are critical for some of the conclusions made. I’ve detailed them below.

Major

• In the introduction, it may be helpful to keep the first paragraph to relevant information in the paper. For example, authors are not looking at long-term social dynamics with changes in group living density, so information like infanticide and cannibalism are not relevant. Instead, literature on hierarchies, novel social interactions, social satiation, etc would be more helpful to set up the present results.

• In the bootstrapping section, it would be helpful to define what a trajectory is defined as. It’s unclear if this refers to tracking a single rat for the entire session or until a goal is met (e.g. reached a social contact, food pellet). If until a goal is met, when was the start and end of a trajectory defined?

• How exactly were clusters defined? Was a group of 6 rats considered 6 for the entire social bout or was it first considered a cluster of 3 until other rats joined? It may be helpful to look at locomotor dynamics between rats that ‘initially’ formed clusters and rats that ‘joined’ clusters.

• Please report full statistics in the results section for each graph/figure

• Authors should account for changes in social vs nonsocial locomotion. For example, chasing/following behaviors vs approach vs movement that does not result in physical contact or proximity to another group

• Authors state that they account for social familiarity, but it’s difficult to interpret where and how this is accounted for. For each grouped scenario (3, 6, 9. 15 rats), were all rats novel to one another, cagemates, or had prior interactions? If familiar, how did interaction change between those conspecifics with larger group sizes? I think I understand that authors state this was based on a ‘baseline’ session to assess familiarity, but did authors log interaction time between conspecifics as an index for familiarity? This influences subsequent behaviors with other conspecifics, so it would be helpful to be as clear as possible and quantify novel vs familiar conspecific interaction times or timing until ‘familiarity’ is achieved based on baseline performance. This should be done outside of the bootstrapping, as ‘real’ and ‘bootstrapped’ experiments differed.

• For the food pellet condition, it is unclear what traveling a great distance means. Do rats travel a greater distance for the pellet, does the food pellet introduce enough novelty that rats increase exploratory movement? How is clustering in larger group conditions accounted for in distance measures here? If a single food pellet went to a single rat, what were the locomotor characteristics of this particular rat during the session before and after? Did this rat consistently receive the pellet across sessions?

• Instead of total clusters, it may be more helpful to examine distance measures between when clusters were formed and time and how clusters influence approach for other conspecifics. This would provide an additional level of detail regarding propensity to socially engage or locomotion for social opportunity.

Minor

• In the methods section, authors may fill in a number for the ‘XX’ in the paragraph discussing the DBSCAN algorithm

• In other ‘words’ vs works in the Bootstrapping section

Reviewer: 2

Comments to the Author

Authors examined how the number of rats affect movement dynamics and clustering, and whether such effects depend on population density or seeking of social interaction, or both. Behaviors were recorded on camera and analyzed using DeepLabCut to assess clustering, speed, and distance. These effects were analyzed in terms of density, familiarity or both. Overall, these results represent an important line of investigation into the ethological factors of motivated behavior in groups. However, there is a need to include more statistics in the manuscript before one can judge the contribution fairly and rigorously.

My major critique is that there is little statistical reporting of any of the Figures in the Results section. Authors state they performed Kolmogorov-Smirnov tests but I could not find these in the Results, just in the Figures. As presented in the Figures, there is no clear description. Authors need to include a text description of the results of the statistical tests. Even in the case that the results are descriptive and do not test specific hypotheses, i.e. “the total number of conspecific approaches generally increased as the number of rats increased” authors should provide means and SEM and/or tests for these in the Results section.

Authors need to improve/increase the size and legibility of axes labels and standardize the look of them across the Figures.

It is unclear how the authors separated the effects observed between conditions as due to either group density or pre-established social familiarity, particularly the latter. Please include more detail. Was housing (i.e., being cage mates) entered into the analysis? If not, authors should consider conducting such an analysis perhaps with regression and/or GLM with familiarity (cage mate or not) as a formal predictor of movement.

Minor

Please delete (line 12, page 5): though we are confident that the results won’t change, but our precision will increase of course.

Associate Editor

Comments to the Author:

Authors will need to remember to include link to data repository, funding details, and acknowledgements - but only upon final acceptance.

**Author response**

We thank the reviewers for their comments. We believe we have addressed all the concerns raised in review, and that our paper is improved as a result. We hope that the editor will find our work suitable for Oxford Open Neuroscience.

\### Reviewer: 1

\#### Comments to the Author

> Authors use an interesting design to test locomotion in varying social

grouping scenarios. Rats are put into an open field and locomotor measures

(distance, approach, etc) were quantified in the presence of 3 to 15 other

conspecifics to investigate movement dynamics with social stimuli. This

paper is interesting but lacks some details that are critical for some of

the conclusions made. I’ve detailed them below.

\#### Major

> In the introduction, it may be helpful to keep the first paragraph to

relevant information in the paper. For example, authors are not looking at

long-term social dynamics with changes in group living density, so

information like infanticide and cannibalism are not relevant. Instead,

literature on hierarchies, novel social interactions, social satiation,

etc would be more helpful to set up the present results.

We have modified the introduction to make it more focused.

> In the bootstrapping section, it would be helpful to define what a

trajectory is defined as. It’s unclear if this refers to tracking a single

rat for the entire session or until a goal is met (e.g. reached a social

contact, food pellet). If until a goal is met, when was the start and end

of a trajectory defined?

We apologize for not being clear; the former is correct. We have changed the word “trajectory” to “path”; we believe it to be a more appropriate term. We now define what we mean by a “path” in the context of this study. Additionally, we have added details to the caption for Figure 2, which shows the paths across time and space for 6 rats.

> How exactly were clusters defined? Was a group of 6 rats considered 6

for the entire social bout or was it first considered a cluster of 3 until

other rats joined?

For this paper, cluster lifetimes were calculated separately for clusters of different sizes. Thus, if a cluster of size 3 was joined by more rats, this larger cluster was considered a new cluster, and the calculated lifetime of the 3-rat cluster came to an end. And to be clear, the clusters were defined on the basis of the largest number of rats they contained therein. As such, a cluster of 6 rats was considered to be 1 cluster of 6 rats (not a cluster of 6, a cluster of 5, a cluster of 4, and 2 clusters of 3, for example). We now make this clear in the text.

> It may be helpful to look at locomotor dynamics between

rats that ‘initially’ formed clusters and rats that ‘joined’ clusters.

The reviewer raises an excellent idea. We have not done so for this study, but plan to do so for our next one, currently in progress. Doing so is not trivial, because we need to modify the clustering algorithm to enable cluster “memory”. We are working on it!

>Please report full statistics in the results section for each

graph/figure

We now add the relevant statistics for all figures, with the exception of the bootstrapped samples- real data comparisons where they are not necessary. As shown in Figures 8 and 9, the difference between the “Real” and the “Ghost” rats is so large that any calculated p-value would approach 0.

> Authors state that they account for social familiarity, but it’s

difficult to interpret where and how this is accounted for. For each

grouped scenario (3, 6, 9. 15 rats), were all rats novel to one another,

cagemates, or had prior interactions? If familiar, how did interaction

change between those conspecifics with larger group sizes? I think I

understand that authors state this was based on a ‘baseline’ session to

assess familiarity, but did authors log interaction time between

conspecifics as an index for familiarity? This influences subsequent

behaviors with other conspecifics, so it would be helpful to be as clear

as possible and quantify novel vs familiar conspecific interaction times

or timing until ‘familiarity’ is achieved based on baseline performance.

Our goal was to sensibly emulate a group of rats that had a semi-natural life, and then examine the impact of varying the size of a group in an open environment on their approach behavior, cluster formation, and locomotion dynamics. As such, all rats in our experiment had familiarity with other rats at experiment-onset by virtue of being housed with others, and they had varying degree of familiarity with the rats they encountered during the study. The rats were housed in triads from weaning until the study, and each triad was included in each condition (3- vs 6- vs 9 vs 15-rat conditions) in a counterbalanced fashion. As such, some triads were first run with another triad, and then went on (the next day) to interact with 2 triads (in the 9-rat condition), and so on. Accordingly, some cohorts experienced co-mingling with 12 rats during their first time in the arena whereas others did so in their 4th time in the arena. Our design was not sufficiently powered to analyze order effects, but we controlled for this possibility by counterbalancing, and we kept the tracking blind to rat-ID (i.e., we did not selectively analyze the differential movement pattern of each rat across conditions, but rather analyzed the collective behavior). More directly to the reviewer’s point, we agree that we did not quantitatively assess familiarity in the current experimental design. Rather, we assessed the rats’ behavior with a sample in which they had the propensity to socially engage with other rats (Real), vs a subsample in which they did not (bootstrapped). We have modified the text accordingly.

> This should be done outside of the bootstrapping, as ‘real’ and

‘bootstrapped’ experiments differed.

We analyzed the “Real” data separately, as we believe that it provides a good estimate of the collective behavior across condition in “natural” rats—that is, rats that can make use of their senses to interact in their environments in the moment, and who have the volition to make decisions with respect to their approach behavior and locomotion at the time of analysis.

Our motivation for the bootstrapping experiment was to examine whether any effects observed in the “Real” rats could be simply explained by a change in “unit” density in each environment. In other words, if rather than rats, we had different numbers of bouncing balls, would the balls move at a different speed, travel different distances, and aggregate differently simply because of there being a different number of them around? The bootstrapping exercise provides an even better control, because it includes the same rats as those in the “Real” experiment (rats that have prior experience), with the main difference that we essentially control for what they can do in-the-moment. That is, the bootstrapped “ghost’ rats do not have the volition, in the moment, to make decisions. So, in a sense, they are analogous to balls (in reference to the aforementioned example) with the twist that they have had “real rat” lives and experiences and have the propensity to ambulate in an environment as such.

We have pondered at length what the ground truth might be for our study. A “true” control might dictate that we house rats individually from weaning and prevent them from interacting with others, and that we then quantify exactly who interacts with whom. In our opinion, doing so would be cruel, since rats are social and we always house them with other conspecifics, and such an experiment would not emulate the activity of real rats or be sensibly generalizable to the real world. And yet, such a control still would not be “pure”, because despite being singly housed, they would have adjacent rats in neighboring cages, and would live in a colony where they can smell, see and hear other conspecifics.

We do have another study in progress in which we first quantify and describe the triadic affiliative behavior of rats prior to their collective experience. This follow-up study should help approximate an answer to the second question above of “who interacts with whom”. The present experiment serves as a sensible baseline for future studies we are currently working on. For the present study, we are comfortable with the “Real” rat data being somewhat noisier, and contaminated by prior social interactions between conspecifics, and believe the bootstrapped samples provide a sensible control to contextualize the “Real” rats’ behavior.

> For the food pellet condition, it is unclear what traveling a great

distance means. Do rats travel a greater distance for the pellet, does the

food pellet introduce enough novelty that rats increase exploratory

movement?

Great question! We believe the data support the latter explanation, since 1. There were few pellets dropped (1 per rat), and 2. The increase in distance traveled was sustained post food drop rather than simply driven by rats reaching the pellet.

How is clustering in larger group conditions accounted for in

distance measures here?

The 2 measures were quantified independently of one another. The arena was large enough that a cluster of 15 rats could technically ambulate (and cover distance) as a cohort.

If a single food pellet went to a single rat, what

were the locomotor characteristics of this particular rat during the

session before and after?

For the present study, we kept the tracking blind to rat ID, so we cannot answer this question for an individual rat. We can only report on patterns of collective behavior. The reviewer raises a very interesting question that we would like to examine in future studies.

Did this rat consistently receive the pellet

across sessions?

Across all sessions, food pellets were dropped after ~4 min of the rats being in the environment. As such, all rats experienced food pellets being dropped in the environment each time they were in session. We explicitly did not drop the food pellets immediately next to any given rat, but in fairness, some rats were in closer proximity simply by virtue of all of them freely ambulating at the time of food drop. Still, the counterbalancing and number of rats included in the design likely prevents the behavior of any single rat driving our observed collective effects.

> Instead of total clusters, it may be more helpful to examine distance

measures between when clusters were formed and time and how clusters

influence approach for other conspecifics. This would provide an

additional level of detail regarding propensity to socially engage or

locomotion for social opportunity.

We think this is a great idea, though beyond the scope of the present study. We absolutely plan to perform such analyses in our future studies.

\#### Minor

> In the methods section, authors may fill in a number for the ‘XX’ in the

paragraph discussing the DBSCAN algorithm

We thank the reviewer for catching our oversight and have now added the appropriate number.

> In other ‘words’ vs works in the Bootstrapping section

We thank the reviewer for catching this typo. It is now corrected.

\### Reviewer: 2

\#### Comments to the Author

> Authors examined how the number of rats affect movement dynamics and

clustering, and whether such effects depend on population density or

seeking of social interaction, or both. Behaviors were recorded on camera

and analyzed using DeepLabCut to assess clustering, speed, and distance.

These effects were analyzed in terms of density, familiarity or both.

Overall, these results represent an important line of investigation into

the ethological factors of motivated behavior in groups. However, there is

a need to include more statistics in the manuscript before one can judge

the contribution fairly and rigorously.

We are pleased that the reviewer thinks our experiment is worthwhile and agree that the first version of our manuscript was lacking in statistical analysis. We now include statistical tests and descriptive measures for all our findings.

> My major critique is that there is little statistical reporting of any of

the Figures in the Results section. Authors state they performed

Kolmogorov-Smirnov tests but I could not find these in the Results, just

in the Figures. As presented in the Figures, there is no clear

description. Authors need to include a text description of the results of

the statistical tests. Even in the case that the results are descriptive

and do not test specific hypotheses, i.e. “the total number of conspecific

approaches generally increased as the number of rats increased” authors

should provide means and SEM and/or tests for these in the Results

section.

We agree with the reviewer. We now include relevant statistical measures for all our findings.

> Authors need to improve/increase the size and legibility of axes labels

and standardize the look of them across the Figures.

We agree with the reviewer and have modified our figures to this effect.

> It is unclear how the authors separated the effects observed between

conditions as due to either group density or pre-established social

familiarity, particularly the latter. Please include more detail. Was

housing (i.e., being cage mates) entered into the analysis? If not,

authors should consider conducting such an analysis perhaps with

regression and/or GLM with familiarity (cage mate or not) as a formal

predictor of movement.

We added more details to describe how the study was carried out, including specifics of the housing conditions. We also included additional information to explain how we contextualized our findings based on our bootstrapping results.

\#### Minor

> Please delete (line 12, page 5): though we are confident that the results

won’t change, but our precision will increase of course.

Of course! We apologize for the confusion. We were trying to imply that once we had done the larger number of bootstrapping replications, that the sentence would be deleted. In other words, it was intended for the reviewers, not the final audience.

\### Associate Editor

Comments to the Author:

Authors will need to remember to include link to data repository, funding

details, and acknowledgements - but only upon final acceptance.

This information is ready to be uploaded to our public lab repository upon acceptance.

**OXFNSC-2023-017.R1 - Effects of group size on movement patterns and clustering dynamics in rats**

**Reviewer 1**

Comment to the Author

The authors have addressed all of my concerns.

**Reviewer 2**

Comments to the Author

The authors have addressed all of my concerns regarding the statistical reporting and I find this version much improved. However, I still find that the axes labels in the Figures as non-standardized throughout (different font sizes and legibility- especially Figures 5 and 6 which have tiny font). This should be corrected for the final version.

**Decision letter**

07-Mar-2024

Dear Dr. Monfils,

It is a pleasure to accept your revised manuscript entitled "Effects of group size on movement patterns and clustering dynamics in rats" in its current form for publication in the Oxford Open Neuroscience. The comments of the reviewers who reviewed your manuscript are included at the foot of this letter.

Please note that this journal operates with transparent peer review. This means that the full peer review history of your article will publish online alongside your article. This includes reviewer comments, editor decision letters, and your author responses.

Next steps

You will receive an email from no-reply@scipris.com within roughly one week. This is your invitation to sign up for an account with SciPris, Oxford University Press’ author portal hosted by Aptara. You will need to create an account if you do not already hold one. Please register or log into your account and follow the online instructions which will guide you through signing your licence and paying the APC. The email and the portal have clearly signposted support options if you need any help during this process.

Please note that SciPris is a completely different system from ScholarOne, so your credentials to submit your manuscript here will not work there. Once you’ve created a SciPris account, you will be able to use it whenever you publish with Oxford Open Neuroscience or any OUP journal. Please note that OUP will only ever request payment for applicable fees be made via SciPris or to an OUP bank account. If you ever have concerns about the legitimacy of a request, please do not hesitate to contact a customer services agent via the SciPris portal or directly via oupsupport@scipris.com.

Thank you for your excellent contribution. On behalf of the Editors of the Oxford Open Neuroscience, we look forward to your continued contributions to the Journal.

Sincerely,

Dr. Alicia Izquierdo

Senior Editor, Oxford Open Neuroscience

aizquie@psych.ucla.edu

Reviewer: 2

Comments to the Author

The authors have addressed all of my concerns regarding the statistical reporting and I find this version much improved. However, I still find that the axes labels in the Figures as non-standardized throughout (different font sizes and legibility- especially Figures 5 and 6 which have tiny font). This should be corrected for the final version.

Reviewer: 1

Comments to the Author

The authors have addressed all of my concerns.

Associate Editor

Comments to the Author:

Please standardize the font size for all Figures in the final version.
